# Integrated Automatic Optical Inspection and Image Processing Procedure for Smart Sensing in Production Lines

**DOI:** 10.3390/s24051619

**Published:** 2024-03-01

**Authors:** Rong-Qing Qiu, Mu-Lin Tsai, Yu-Wen Chen, Shivendra Pratap Singh, Cheng-Yao Lo

**Affiliations:** 1 Institute of NanoEngineering and MicroSystems, National Tsing Hua University, Hsinchu 300044, Taiwan; winghingyao@gapp.nthu.edu.tw (R.-Q.Q.); n0022057@gapp.nthu.edu.tw (Y.-W.C.); shivendrasingh@gapp.nthu.edu.tw (S.P.S.); 2 Department of Power Mechanical Engineering, National Tsing Hua University, Hsinchu 300044, Taiwan; asd81673413@gmail.com

**Keywords:** automatic optical inspection, image processing, line edge roughness, machine learning, printed circuit board, spiral antenna

## Abstract

An integrated automatic optical inspection (iAOI) system with a procedure was proposed for a printed circuit board (PCB) production line, in which pattern distortions and performance deviations appear with process variations. The iAOI system was demonstrated in a module comprising a camera and lens, showing improved supportiveness for commercially available hardware. The iAOI procedure was realized in a serial workflow of image registration, threshold setting, image gradient, marker alignment, and geometric transformation; furthermore, five operations with numerous functions were prepared for image processing. In addition to the system and procedure, a graphical user interface (GUI) that displays sequential image operation results with analyzed characteristics was established for simplicity. To demonstrate its effectiveness, self-complementary Archimedean spiral antenna (SCASA) samples fabricated via standard PCB fabrication and intentional pattern distortions were demonstrated. The results indicated that, compared with other existing methods, the proposed iAOI system and procedure provide unified and standard operations with efficiency, which result in scientific and unambiguous judgments on pattern quality. Furthermore, we showed that when an appropriate artificial intelligence model is ready, the electromagnetic characteristic projection for SCASAs can be simply obtained through the GUI.

## 1. Introduction

Printed electronics and printing technologies have been receiving attention because they exhibit a balance between quality and cost. Printed circuit boards (PCBs) have been widely used in various applications. To ensure the fabrication quality of PCBs, researchers have proposed various methods to quantify pattern integrity by evaluating their visual appearance using automatic optical inspection (AOI) procedures [1,2]. Line width roughness (LWR) [3] and line edge roughness (LER) [4] have been adopted, which facilitate the evaluation with scientific and quantified indicators. Process instability can thus be determined or suspected by identifying geometric changes [5]. In addition, some characterizations can be projected as a part of quality control, quality assurance, or early warning [6].

However, the aforementioned methods have the shortcomings of the inability to analyze complex patterns with low efficiency [3] and noticeable errors [7]. They also do not fully support dimension measurements and fail to connect geometric variations with electromagnetic (EM) properties [4]. Furthermore, most procedures in these methods require manual operations and no unified standards are set for image differentiation, preprocessing, data induction and calling, and parameter adjustment through AOI; therefore, analyses based on these existing methods are laborious and time-consuming. Although data acquisition can be performed automatically in part, the analyses of large quantities of samples are still challenging for users without an engineering background. For example, when the images of interest captured from the PCB samples are complicated, users have to refine parameters, such as focus and contrast, to fulfill specific requirements. Users also have to convert the images of interest with proper settings (such as grayscale) before the analyses can be performed. Furthermore, the results can be erroneous when the alignments for correcting magnification, shift, rotation, and tilt are not properly conducted with a universal solution for different samples [4]. In addition, as for the technology that combines image processing and AOI, most of the current industrial applications are to skeletonize the circuit of the PCBs before determining whether the circuit is complete, based on the image processing results. In those process steps, the precision requirements for image processing are relatively low and the requirements are relatively loose. In short, existing methods cannot provide a single solution to support smart manufacturing with operational efficiency in handling related procedures in a standard and integrated manner.

Therefore, this paper proposes the development of an integrated AOI (iAOI) system and procedure to minimize manual handling steps, and provide a turn-key and one-step solution. In the proposed system and procedure, several standardized steps for image processing and data analysis were designed, and a graphical user interface (GUI) was developed. The proposed procedure contains five analysis steps and starts with image registration as the first step, where the image of interest obtained from the PCB sample can be imported to the computer from an existing file or a camera that operates instantly. Subsequently, proper threshold settings should be performed as the second step to ensure that good contrasts appear between the target and non-target locations on the image of interest. To further enhance sharpness and remove noise, an image gradient should be conducted as the third step. The markers that are generally arranged close to the image of interest for alignment should be correctly positioned in the fourth step before the geometric transformation of the image can be executed by the computer in the fifth (the last) step. These ensure unified operations and show operational simplicity to general users who are unfamiliar with engineering interfaces.

**Figure 1:**
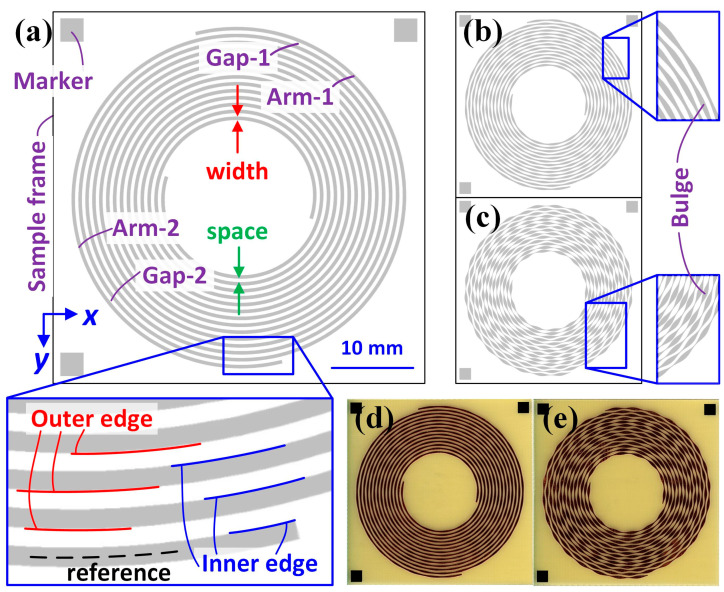
The SCASA appearance in the design with a BA of (**a**) 000, (**b**) 030, and (**c**) 075, and in PCB with a BA of (**d**) 015 and (**e**) 060. Note that the reference line (only partially illustrated in dash in (**a**)) is located at the center of the arm.

To demonstrate the effectiveness of the proposed iAOI system and procedure, self-complimentary Archimedean spiral antennas (SCASAs) fabricated using standard PCB fabrication procedures were introduced as samples, in which pattern variations in terms of bulge amplitude (BA) appeared in both arms of the SCASAs (Figure 1) [8]. By analyzing the SCASAs through the proposed iAOI system and procedure, the image of interest and related data can be acquired effectively and efficiently compared with existing methods. These data can be used to establish a database for EM characterization and its prediction using artificial intelligence (AI) through machine learning, which has not been supported in similar solutions.

## 2. Workflow

**Figure 2:**
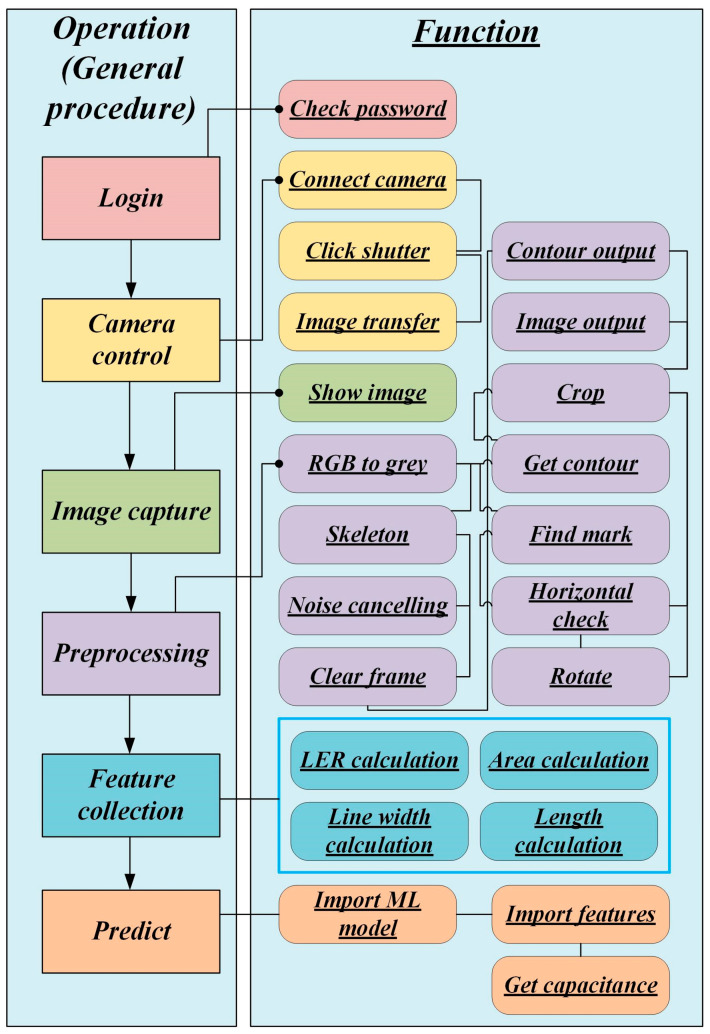
The workflow of the proposed iAOI procedure. Note that functions are listed in part.

In this study, an iAOI system with a camera and backlight unit (BU) hardware in an opaque box was used as a module. Nevertheless, for the iAOI procedure, the GUI was created by Python with application programming interfaces (APIs), such as DigiCam [9], Tkinter [10], OpenCV [11], and NumPy [12], to control the camera in the demonstrative module, perform image processing, and evaluate features with several operations in a workflow shown in Figure 2. The complete script of Python was written through C (programming language); thus, the stability and efficiency were reliable. Meanwhile, Python has become more and more popular because of its short development cycle; therefore, industries and academia are willing to develop APIs on Python. Consequently, this work adopted Python programmed through C, in which various APIs existed in this work. We believe that the developed Python script through C was reliable in this work.

**Figure 3:**
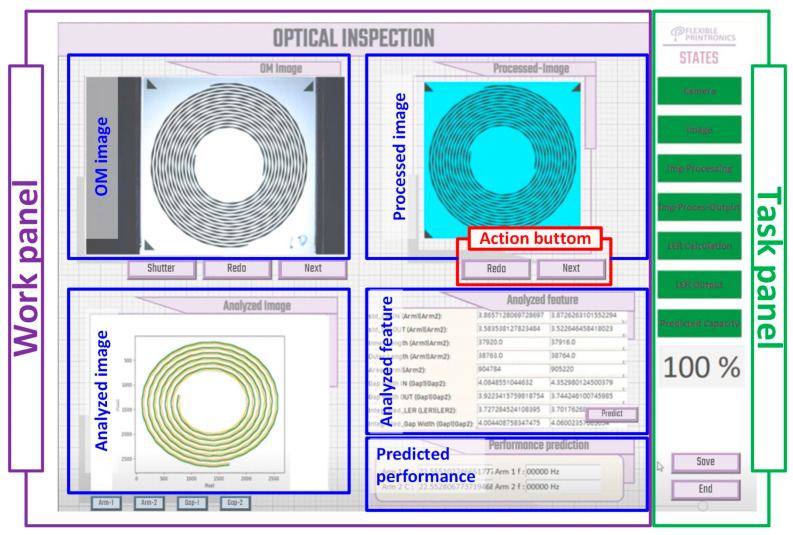
The GUI of the proposed iAOI system.

From the GUI, users may initiate the iAOI system with a login page by performing the operation *Login*, which prompts the password. (In this work, operations are described in *italic* for clarity.) In addition to *Login*, five other operations exist: *Camera control, Image capture, Preprocessing, Feature collection, and Predict*. The GUI (Figure 3) appeared in a work panel with five windows to indicate the conditions of the corresponding image of interest or data after processing. The Optical Microscope (OM) image, processed image, and analyzed image window display the image of interest captured through the camera, processed through the proposed procedures, and analyzed using features, respectively. (In this work, windows in the GUI panels are underlined for clarity.) The analyzed feature and predicted performance window display the quantified data extracted from and projected onto the analyzed image, respectively.

To display the progress, a task panel with seven status indicators was designed in the GUI, which shows the progress of the corresponding iAOI procedures as a percentage. The seven indicators refer to (1) the readiness of the camera, (2) the readiness of the OM image window, (3) the progress of *Preprocessing*, (4) the readiness of the Processed image window, (5) the progress in *Feature collection*, (6) the readiness of the Analyzed feature window, and (7) the progress in operation Predict and readiness of the Predicted performance window.

In the GUI, the user may press one of the action buttons designed for the windows in the work panel. For example, in the OM image window, when the “Shutter” is clicked, the operation *Camera control* is started, and the signal is sent between the camera and computer through the USB interface. (In this work, actions attached to the windows in the work panel were “bracketed” for clarity.) The camera captured the image, sent it back to the computer, and displayed it on the OM image window through the operation image capture. If users are not satisfied with this image, it can be cleared by clicking “Redo”. Otherwise, this image is sent for adjustment by clicking “Next,” which initiates the operation *Preprocessing*.

In *Preprocessing*, several functions for gray scaling and noise cancellation will be conducted to refine the image to the proper condition for further analyses [13], which will be explained later. Similar to the actions that appear in the OM image window, “Redo” provides a chance for users to remove the existing image and re-capture the image from the camera. Otherwise, the adjusted image will be passed for analyses by pressing “Next,” which initiates the operation *Feature collection* with several functions for advanced adjustment and coordinate collection, which will also be described later.

In the Analyzed image window, users may opt to display and focus on individual contours on the arms or gaps; thus, four action buttons of Arm-1, Arm-2, Gap-1, and Gap-2 are assigned. In this study, both the outer and inner LERs on the two arms of the SCASAs were collected (Figure 1a). These LERs can be used to obtain arm lengths, areas, and gaps between the two arms. Finally, the features were summarized and displayed in the Analyzed feature window. To this point, the physical characteristics are qualified, and comparisons can be achieved if the expected values (for example, the designed dimensions) exist.

In addition to feature analyses, this study aimed to predict the characteristics of SCASAs. Consequently, the quantified values of the features are fed into the developed AI model [4], and the predictions are shown in the Predicted performance window when the action “Predict,” designed for the Analyzed feature window, is clicked. In the demonstration, the capacitance between the two arms was studied, and the users exported the predictions or terminated this GUI by clicking “Save” or “End” in the task panel, respectively.

Regardless of the image, the aforementioned workflow supports the general operation of feature characterizations provided that the corresponding analysis algorithms and AI models are ready. Consequently, typical and universal steps are described below, which also support the demonstrative SCASAs adopted in the present study. In this study, five analysis steps, including image registration, threshold setting, image gradient, marker alignment, and geometric transformation, were considered, which sequentially lead the image of interest to an appropriate one before characteristics, such as LER, can be analyzed or quantified. The detailed operating procedure was described in the Supplementary File (composition of the GUI).

## 3. General Procedure

### 3.1. Image Registration

Image registration is the process in which the image of interest is captured by the camera and delivered to the computer [14]. However, various hardware settings and software operations may cause different image conditions, which in turn lead to different analysis results [15]. Consequently, hardware settings, such as BU contrast, should be considered. Additionally, because the control on the camera is performed through the GUI, related functions of *Click shutter*, *Image transfer*, and *Display image* will be conducted in operation *Camera control.*

By realizing image registration through the automated and standardized steps proposed in this work, users can receive the required image quality and avoid instabilities arising from manual operations and calibrations, regardless of the sample. Furthermore, these automated steps improve overall efficiency, which will be discussed later.

### 3.2. Threshold Setting

The image of interest underwent several functions in the operation *Preprocessing* to meet the specifications defined for analyses. These functions are designed for image binarization and thresholding [16,17], which extracts the target area (for example, the arm or gap shown in Figure 1a) from the image of interest by observing its grayscale.

In API OpenCV, two categories of simple and adaptive binarization exist. Simple binarization is a straightforward option because it removes major noise in most cases. However, the appropriate sharpness of the contours of the pattern is also expected when performing the analyses. Accordingly, adaptive binarization that shows additional control of the parameters (such as “*block size*” and “*non-zero value*”) in the functions was also adopted. (In this work, API parameters are described in “*bracketed italic*” for clarity). Among the various maneuverable parameters in these two binarizations, “*maxValue*” was the key to the present study, which was set to integers between 0 and 255.

**Figure 4:**
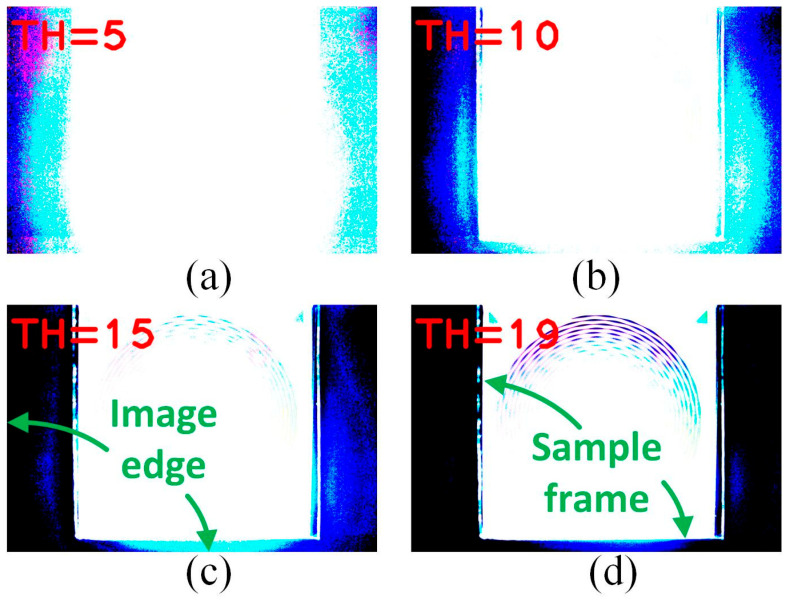
An example of a simple binarization-processed image with threshold (TH) settings of (**a**) TH = 5, (**b**) TH = 10, (**c**) TH = 15, and (**d**) TH = 19 from the BA030 sample.

**Figure 5:**
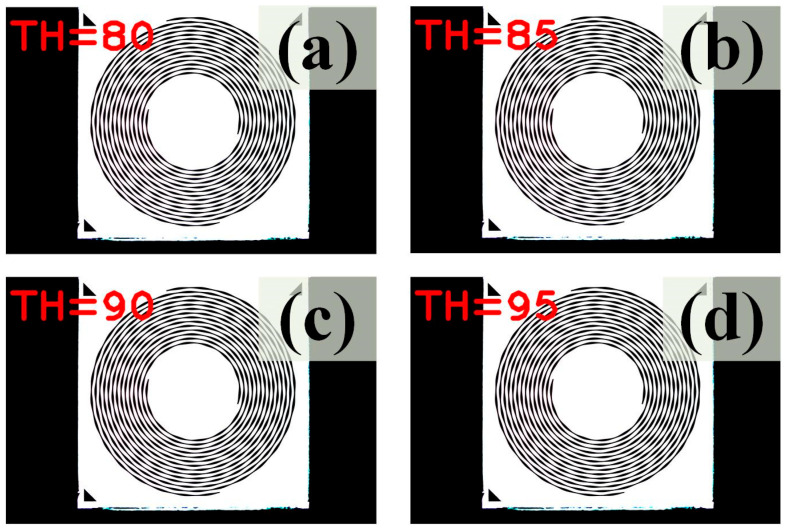
An example of an adaptive binarization-processed image with threshold (TH) settings of (**a**) TH = 80, (**b**) TH = 85, (**c**) TH = 90, and (**d**) TH = 95 from the BA030 sample.

When “*maxValue*” was set to 0–56, most of the areas on the image showed excessive backlight because there were only limited grayscales (Figure 4). These results cannot effectively distinguish pattern contours from the background [18]. However, when “*maxValue*” was set to 57 to 255, some noise appeared, and reasonable and acceptable results were found. After several trials, only when “*maxValue*” was set to 83 to 93, appropriate sharpness and clear contours were obtainable for analysis [19]. Consequently, a “*maxValue*” of 85, which showed the most stable appearance regardless of the BA was selected (Figure 5). At this point, simple binarization has already removed the noise in the image of interest. However, as aforementioned, adaptive binarization was continuously applied to further enhance the appearance of the contour.

By applying a “*maxValue*” of 0 to 89 and 116 to 255 through adaptive binarization to the image after simple binarization (a “*maxValue*” of 85), broken contours were found, which cannot be used for analyses. After several trials, a “*maxValue*” of 90 to 115 resulted in clear borderlines (sample frame shown in Figure 1a) outside the images of interest, indicating proper conditions for further analysis. Because the borderline should be sharp and the image around the markers should not display any noise, a “*maxValue*” of 90 was determined to be the best condition for adaptive binarization.

### 3.3. Image Gradient

After the thresholds of the simple and adaptive binarizations were determined, a gradient was applied to obtain the boundary of the patterns because each pixel had a grayscale between 0 (black) and 255 (white). The greater the difference in grayscale between adjacent pixels, the more noticeable the boundary. Because the change in grayscale in adjacent pixels can be the judgment for the boundary, algorithms of **Sobel** [20], **Scharr** [20], **Laplacian** [21], and **Canny** [16] were considered, which showed respective benefits. (In this study, the algorithms are marked in **bold** for clarity.)

**Sobel** was designed to extract thick and continuous contours; thus, it supports patterns with simple shapes and sharp edges. **Scharr** is an adjusted version of **Sobel** that aims to obtain a cleaner image with an extended kernel. However, both **Sobel** and **Scharr** sacrifice the resolution of complex patterns, showing deficiencies in the proposed iAOI procedure. Similarly, **Laplacian** cancels noise; however, it occasionally removes expected contours by erroneously judging them as noise, thereby displaying incorrect results.

**Figure 6:**
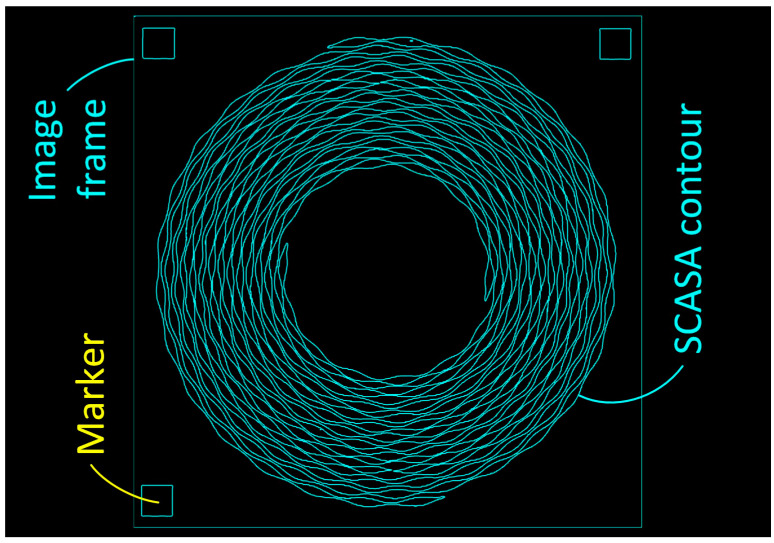
The contours of the BA075 SCASA, markers, and sample frame after simple (threshold of 85) and adaptive (threshold of 90) binarizations, and image gradient.

However, **Canny** is a composite edge detection algorithm that combines Gaussian filter, gradient detection, non-maximum suppression, and boundary judgment, which exhibits the advantages of low error rate (background and pattern can be separated with high contrast), accurate positioning (the marked edge is close to the actual edge), and high resolution (fine contours are generated) over the other three algorithms in this work in the tolerance viewpoint. Consequently, this work introduced **Canny** as the algorithm for the image gradient, and the resultant image (after threshold setting) is shown in Figure 6.

### 3.4. Marker Alignment

To analyze the contours of the image, the image frame should be removed, and a function *Clear frame* is created and performed before alignment is conducted. Subsequently, a function *Find mark* is created to obtain the relative coordinates of the markers. (In this paper, functions are described and *underlined in italic* for clarity.) Because markers are key to alignment, effective and practical markers are required [17].

**Figure 7:**
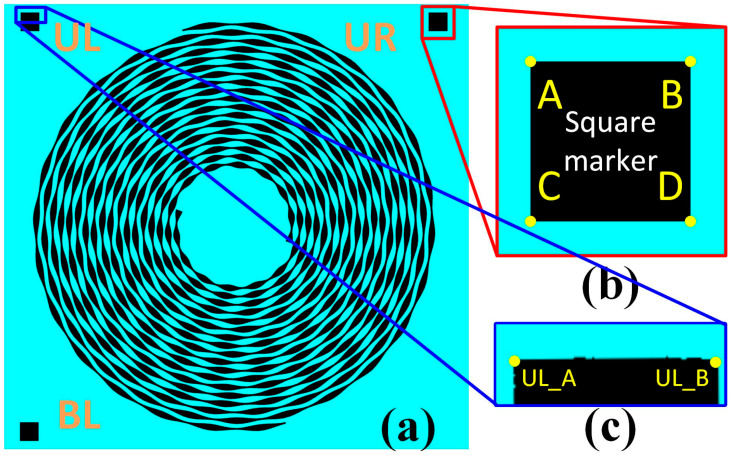
The high-definition images of the (**a**) location, shape, and relative position of three square markers in one SCASA, and (**b**) their corner definitions with (**c**) partial magnification in practice of a BA045 sample.

**Figure 8:**
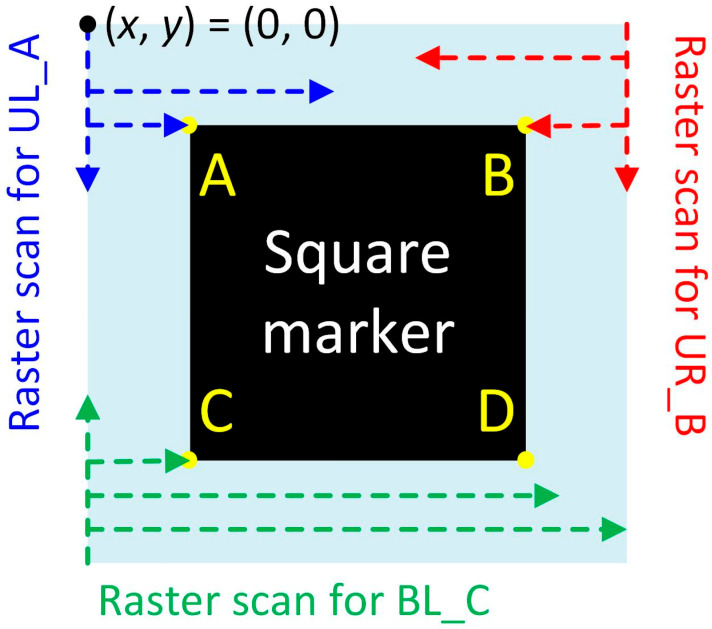
The explanation of the raster scan procedure on the corners A, B, and C at their corresponding markers.

In this work, three square markers located at the three corners (UL at the upper left, UR at the upper right, and BL at the bottom left) of the SCASA sample were originally adopted (Figure 7a). Each square marker had four corners and was named A, B, C, and D, as shown in Figure 7b. Before alignment, the coordinates of any specific corner should be known through a raster scan, which starts from the upper left corner of the image in the format (*x*, *y*) (Figure 8) and has been widely adopted in similar studies [22]. In this work, Cartesian coordinates were used, where x represents the pixel number in the horizontal direction from left to right, and y represents the pixel number in the vertical direction from top to bottom.

In the function *Find mark*, the coordinates of the corners UL_A, UR_B, and BL_C were expected to be accurately obtained (Figure 8). Accordingly, the row-by-row raster scan for corner UL_A started from the left to a point located at the middle of the image with *y* = 0 to avoid erroneous coordinate collection from marker UR. The function stopped at a point with a black pixel that showed (R, G, B) = (0, 0, 0). If there were no black pixels in a row, an identical scan was repeated with *y* = 1. For corner UR_B, the raster scan also started row by row, however, from the right to a point located in the middle of the image to avoid erroneous coordinate collection from the marker UL. Similarly, the function stopped at the corresponding black pixel in the marker UR. Because marker BL is located on the same side as marker UL, the procedure of collecting the corner BL_C coordinate was identical to that of corner UL_A; however, the row-by-row raster scan started from the bottom. These coordinates are used for rotation and shift alignment later, and the image theoretically fulfills the requirements for geometric transformation after alignment.

### 3.5. Geometric Transformation

**Figure 9:**
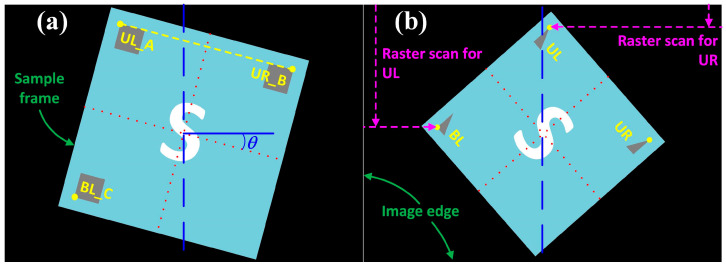
The illustrative explanation of the rotational and shift alignment of the target image (S) with (**a**) square and (**b**) triangle markers to different extents of rotation. Dotted and long dashed lines are references for the sample and image center, respectively.

After locating the markers with their coordinates, the target pattern should stand upright for analysis. Thus, a function *Rotate* that adjusts the image into the correct orientation was prepared in this work. By considering the center of the PCB sample as both the rotational center and the center of the adjusted image, and by selecting the coordinates of corners UL_A and UR_B; *θ* and the rotation of the image (clockwise or counterclockwise) were understandable (Figure 9a).

In addition to the rotation, the horizontal or vertical shift of the image should be known, which can also be obtained through trigonometry and *θ*. Finally, the function Rotate adjusts the image not only into the correct orientation but also the location by rotating and shifting it, respectively. To this point, the image was ready for feature analysis, and demonstrations of SCASAs made on PCB samples were conducted as described below.

## 4. Demonstration

### 4.1. Hardware

**Figure 10:**
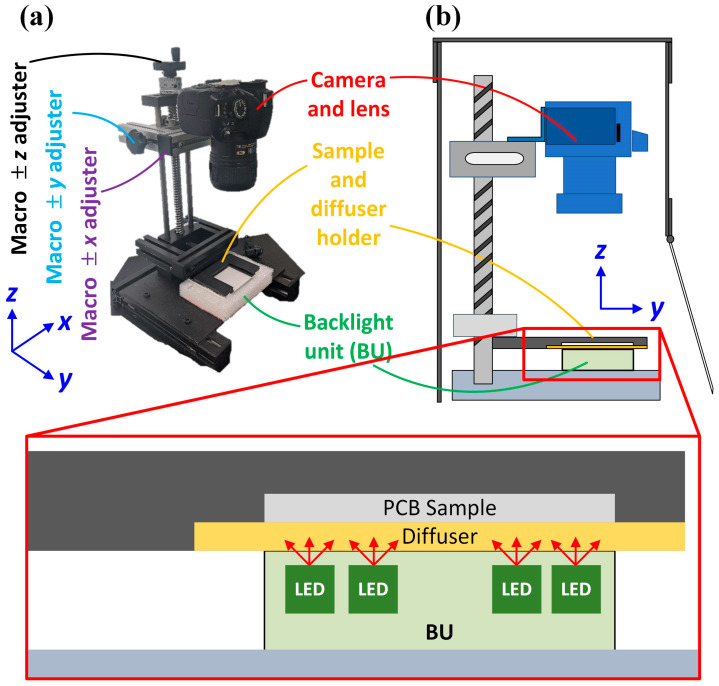
The demonstrative image-capturing module in (**a**) practice and (**b**) design with BU detail. The module was completely covered by another opaque housing and (**b**) was not drawn to scale.

Although typical AOI systems contain basic image-capturing modules, such as a charge-coupled device controlled by a computer, this study adopted a commercial single-lens reflex (SLR) camera (Nikon, D3100, Tokyo, Japan) with a lens (Nikon, AF-S Micro NIKKOR 60 mm F/2.8G ED) (Figure 10a). This setup proved that general cameras can support the proposed iAOI system and procedure without the need for advanced equipment, showing support for simple and budget operations.

The environment must be controlled to ensure the authenticity of the image [23]. Consequently, an image-capturing module (opaque box) was designed to avoid ambient interference (Figure 10b). Inside the module, the SLR and its lens were installed on a stand and connected to the computer through a USB for various functions in operation *Camera control*. Furthermore, a vertical adjuster was present, which provided macroscopic movement of the camera. Meanwhile, the lens itself provided microscopic focus optimization through the camera during image acquisition.

In practice, samples are placed above a diffuser (Yongtek, DF-188SF, 0.23 mm-thick), which in turn stands atop the BU composed of surface-mount device (SMD) light-emitting diodes (LEDs), during image capturing to ensure appropriate image quality. Consequently, an adequate diffuser specification was investigated for the BU in this study.

### 4.2. Algorithm

Python was used as the programming language with third party APIs mentioned previously for feature analyses. The logic of integrated LER analysis came from an existing method [4], which quantifies it in a single arm in terms of standard deviation (*σ*) with (1).

(1) $$\mathrm{LER}=\sqrt{\frac{\left(n-1\right)\sigma_{O}^{2}+\left(n-1\right)\sigma_{I}^{2}}{2n-1}},$$

(2) $$\mathrm{LER}=\sqrt{\frac{\left(n-1\right)\sigma_{O1}^{2}+\left(n-1\right)\sigma_{I1}^{2}+\left(n-1\right)\sigma_{O2}^{2}+\left(n-1\right)\sigma_{I2}^{2}}{4n-1}},$$

In (1), *n*, $\sigma_{O}$, and $\sigma_{I}\;$ represent the total number of data points, outer (denoted in superscript as O) LER, and inner (denoted in superscript as I) LER of that arm, respectively. In this study, advanced analyses were expected, in which not only a single arm but also both arms were involved. Consequently, additional information from another arm is required, and the two arms are analyzed simultaneously with (2), where subscripts 1 and 2 represent Arm-1 (or Gap-1) and Arm-2 (or Gap-2), respectively. The mathematical models were detailed in the Supplementary File (image gradient).

As mentioned in Section 2, Python was programmed through C, where various reliable APIs exist. As a result, this work adopted those aforementioned APIs in this work.

### 4.3. Trial and Optimization

#### 4.3.1. Sharpness Enhancement

Because the image contrast influences the analysis, which was decided by the backlight, the uniformity of the backlight resulting from both the LED arrangement in the BU and diffuser efficiency are discussed here. Furthermore, the aim of related studies is to obtain accurate results, which can be evaluated by understanding the difference between the average LERs on the designed (reference) and measured images. Consequently, a minimal value of the average LER difference was expected, which was evaluated simultaneously through the brightness (obtained through the image and in units of lumens (lm)) and diffuser setup (in units of layers).

**Figure 11:**
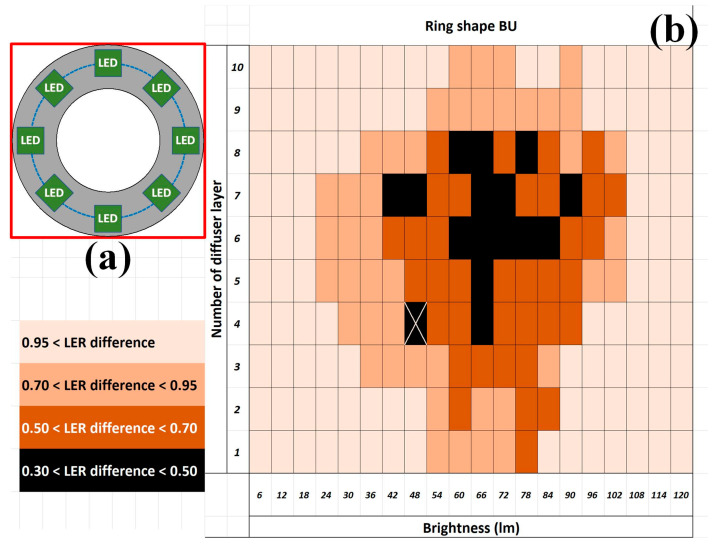
The BU (not to scale) (**a**) illustrative ring shape arrangement from the top and its (**b**) resultant distribution of the average LER difference between the designed and measured images. Note that the minimal average LER difference appearance was marked X and the minimal LER difference is in the unit of pixels.

**Figure 12:**
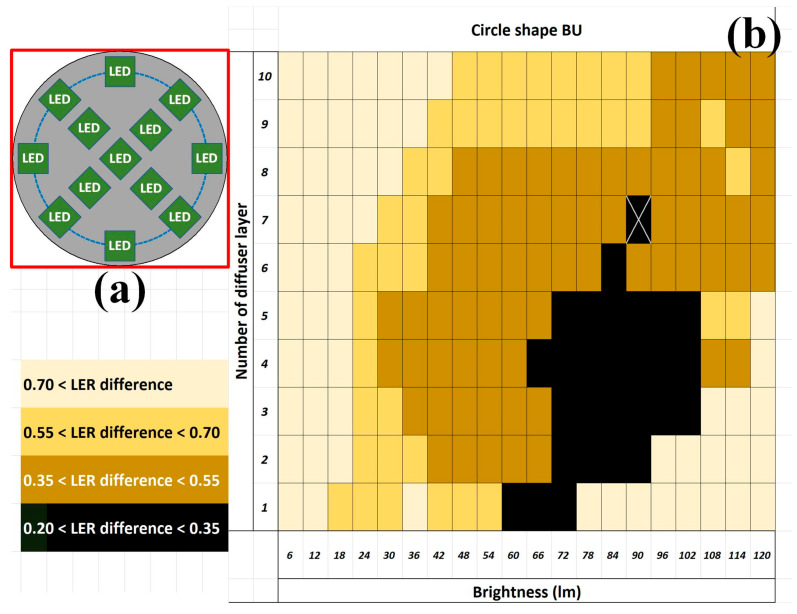
The BU (not to scale) (**a**) illustrative circle shape from the top and (**b**) the resultant distribution of the average LER difference between the designed and measured images. Note that the minimal average LER difference appearance was marked X and the minimal LER difference is in the unit of pixels.

In this study, the BU (405 × 405 mm^2^, identical to the dimensions of the sample frame), composed of LEDs (7 × 7 mm^2^ each) and arranged in a ring shape, was treated as the fundamental setup (Figure 11a), and three arrangement advancements in circle, frame, and square shapes were studied [24]. The results obtained from the ring-shaped BU indicated that the minimal average LER difference between the designed and measured images was smaller than 0.5 pixels when the intensity was 48 lumens and the diffuser contained four layers (Figure 11b). Although this value may not be observable by the naked eye, this work aimed to further suppress it when micrometer- or nanometer-scale patterns are to be analyzed with high-resolution images in other applications. Because the backlight from the ring-shaped BU failed to cover the center of the PCB sample, the BU was modified to a circle shape (Figure 12a). More LEDs were adopted in the BU and thus thicker diffusers would be required to achieve similar brightness. The results from the circle-shaped BU indicated that the minimal average LER difference between the designed and measured images was smaller than 0.35 pixels when the intensity was 90 lumens and the diffuser contained seven layers (Figure 12b). Although the absolute improvement in the minimal average LER difference was limited, the relative improvement (30%) was noticeable.

**Figure 13:**
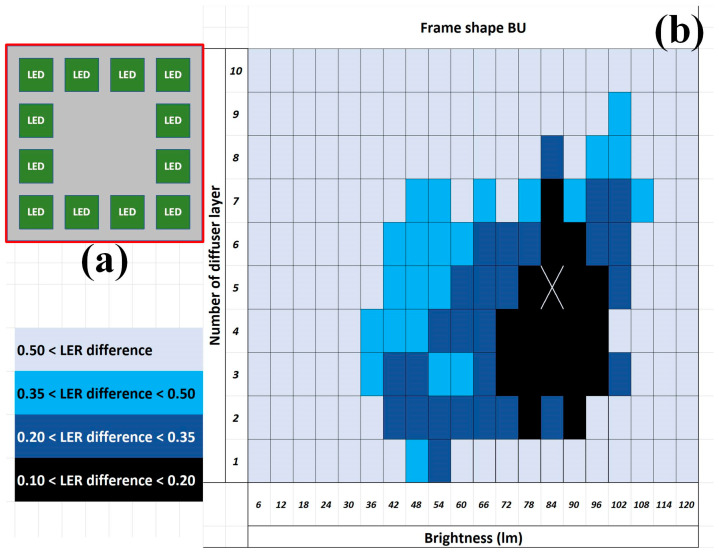
The BU (not to scale) (**a**) illustrative frame shape from the top and (**b**) the resultant distribution of the average LER difference between the designed and measured images. Note that the minimal average LER difference appearance was marked X and the minimal LER difference is in the unit of pixels.

To further suppress the minimal LER difference, a BU with frame-shape-arranged LEDs was considered (Figure 13a) because the markers, which were key components for alignments and analyses as aforementioned, were located at the corners of the PCB samples. The results from the frame shape BU indicated that the minimal average LER difference between the designed and measured images was smaller than 0.2 pixels (60% and 43% improvement from the ring- and circle-shaped BU, respectively) when the intensity was 84 lumens and the diffuser contained five layers (Figure 13b).

**Figure 14:**
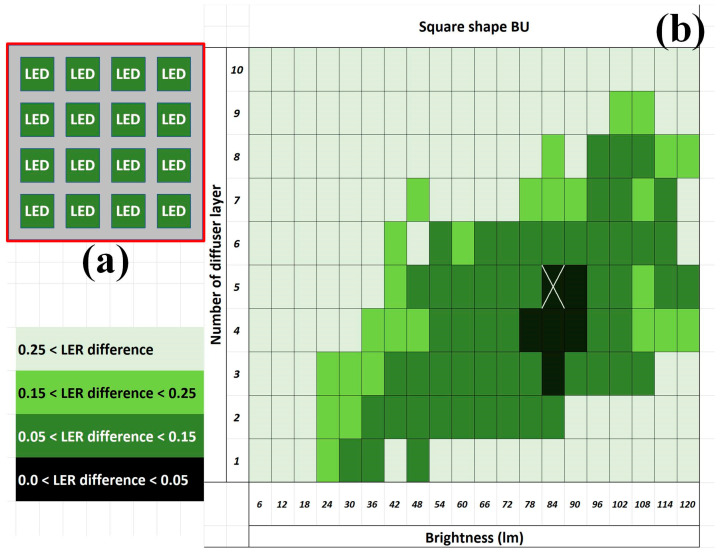
The BU (not to scale) (**a**) illustrative square shape from the top and (**b**) the resultant distribution of the average LER difference between the designed and measured images. Note that the average minimal LER difference appearance was marked X and the minimal LER difference is in the unit of pixels.

Based on the results obtained with the circle-shaped BU, LEDs were filled into the central part of the frame-shaped BU as a square-shaped BU (Figure 14a) to further explore the effectiveness of the suppression on the minimal average LER difference. The results from the square-shaped BU indicated that the minimal average LER difference between the designed and measured images was as small as 0 pixels when the intensity was 84 lumens and the diffuser contained five layers (Figure 14b). Because the minimal average LER difference achieved 0 pixels in the square-shaped BU, which could not be further improved, this work adopted the square-shaped BU for the analyses.

#### 4.3.2. Marker Modification

**Figure 15:**
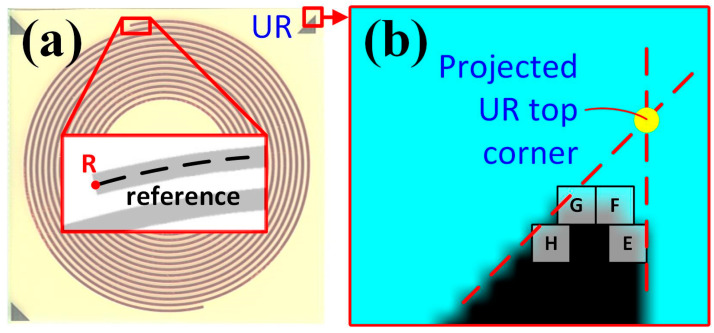
The triangle marker in (**a**) the PCB sample (BA000) and its (**b**) processed image (in part) with explanation on the unexpected coordinate collection at the top corner.

Previously, square markers were explained, which are widely used in various applications, such as quick response codes [25]. Because one square marker has four corners, and the corners already show geometric distinctions, it could theoretically be identified for coordinates. However, the image to be analyzed generally cannot display sharp corners or regular edges owing to various reasons in the AOI (such as inappropriate contrast) when the resolution is high. Consequently, the square markers can only be recognized as arbitrarily enclosed areas (Figure 7c) instead of meaningful shapes for alignment, particularly when the image is rotated. For example, corner UL_B, instead of UL_A, is collected in a counterclockwise rotated image. Similarly, UR_A is collected in a clockwise-rotated image instead of UR_B. Consequently, erroneous alignment actions can be made using square markers. Furthermore, if the coordinates obtained from the function *Find mark* are incorrect, consecutive functions, such as *Rotate* (for alignment), *Shift* (for alignment), and *Crop* (for frame removal), will be negatively affected [26]. As a result, the square marker should be modified into a shape that does not exhibit these potential issues. Because triangle markers show higher efficiency and accuracy with a lower error rate, they were adopted in this study (Figure 15a).

Theoretically, because there is only one point (pixel) at the top corner of the triangle marker, the issue mentioned before does not occur regardless of clockwise or counterclockwise image rotation. However, inappropriate contrast still results in rounded corners in the triangle markers at high resolution (Figure 15b). These rounded corners may still lead to incorrect coordinates for analysis, although their impact has been extensively suppressed by square markers. For example, none of the E, F, G, or H pixels are expected to be the top corner of the marker UR. However, point F is still recorded through the function, which represents an incorrect coordinate for the alignment. To address this issue further, a solution that projects the correct locations of the top corner in the triangle marker from the image was proposed.

In Figure 15b, all points located near the rounded top corner with (R, G, B) = (0, 0, 0) were scanned and recorded in coordinates. With these coordinates, the hypotenuse and vertical sides of the triangle marker were determined. Consequently, the intersection of the prolonged hypotenuse and vertical side leads to the theoretical coordinate of the top corner of the triangle marker, which is used for related image processing and analysis functions. In addition, among all the right-angle triangles, the top corner with an angle of 45° was selected for the following reasons.

When the top corner is smaller than 45°, the markers support better rotational alignment regardless of the orientation, because the tolerance limit is set by a condition in which the hypotenuse of the triangle is parallel to the *x*-direction. However, before the hypotenuse of the triangle marker is rotated parallel to the *x*-direction, the marker UL is already moved to a location in the right part of the image in the example of clockwise rotation (Figure 9b). Therefore, the expected UR coordinates are erroneously collected through the marker UL. A similar incorrectness also occurs in counterclockwise rotated images. Considering that a top corner larger than 45° shows less tolerance and practical handling (the maximum rotational misalignment will never surpass 45° in all cases); this work adopted a 45° right-angle triangle as the shape of the markers.

To this point, rotational alignment (image stands upright) was performed. However, because the projected top corner of the triangle marker can still exhibit shifts (along the *x*- or *y*-direction), although the image stands upright, a second alignment that helps eliminate the offset between the processed image and the reference pattern is required.

#### 4.3.3. Alignment Refinement (Second Alignment)

**Figure 16:**
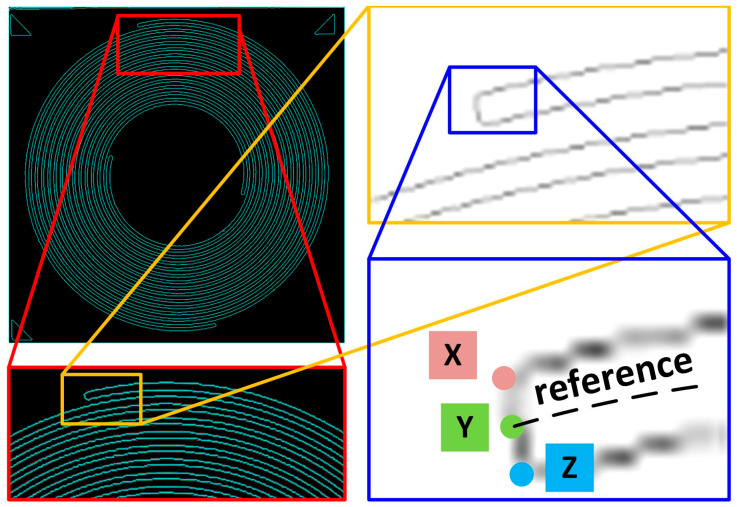
The explanation of the determination of point Y and the reference line in the image of interest. X, Y, and Z represent the end point of the outer contour, reference line, and inner contour of the image, respectively, regardless of their color legends.

The LER analysis in this work was based on the quantification of the distance between the reference lines on the target pattern (Figure 1a) and on the image of interest, with their absolute locations in Cartesian coordinates. The reference line on the target pattern can be correctly determined from the design; however, the reference line on the image of interest is calculated through both edges of one arm. An offset exists between the reference lines of the actual and calculated coordinates in the image of interest, and a fair comparison can only be made when the two lines are completely superimposed. To eliminate this offset, the coordinates of the endpoint on the image of interest must be identified. The coordinates of point Y (Figure 16) on the image can be obtained through point X (or point Z) and a line width in the image with a specific BA; however, this method cannot be applied to other images with different BAs because the line width varies in practice. Consequently, a method that determines the coordinates of point Y through points X and Z was used.

First, a limited scanning area was determined, in which the absolute coordinates of the contours on the image of interest were collected. With an identical scanning method (function *Find mark*) used for marker identification, points X and Z lead to point Y. Here, the function *Find mark* was revised with a different raster scan sequence, in which point X was collected by scanning from left to right and from the top to bottom of the determined area. However, point Z was collected by scanning from left to right, but from the bottom to the top of the determined area. To this point, the coordinates of point Y, point R on the reference line (Figure 1a), and their offsets are known.

Second, because all contour points on every individual edge are continuous and the image of interest is stood upright after alignment, it is reasonable to shift the complete image of interest with a unified offset for correct analysis.

## 5. Result and Discussion

**Figure 17:**
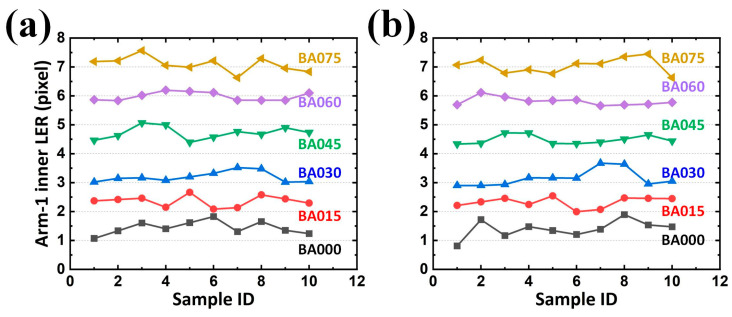
Analyzed LERs on the (**a**) inner and (**b**) outer edge of Arm-1 along various BAs in 10 PCB samples.

**Figure 18:**
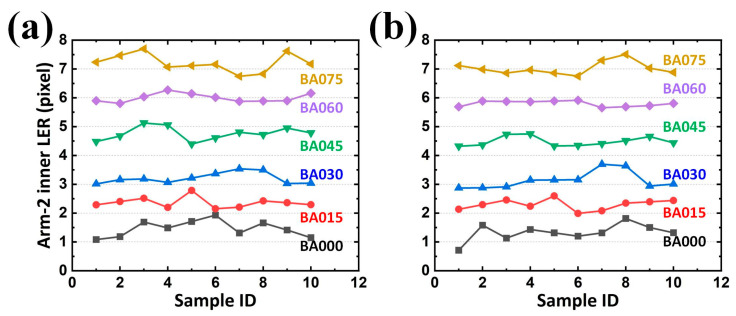
Analyzed LERs on the (**a**) inner and (**b**) outer edge of Arm-2 along various BAs in 10 PCB samples.

Because each sample for analysis contained two arms (or two gaps), and each arm (or gap) contained two contours, one analyzed image returned four LERs on the developed GUI regardless of their designed BA. Cumulatively, ten samples for each BA were analyzed in this study. Statistically, the results revealed that an increasing average LER existed along the enlarged BA, reflecting the expected tendency. Although the target LER for BA sets of 000, 015, 030, 045, 060, and 075 in the inner contour of Arm-1 were 0, 1.655, 2.894, 4.304, 5.599, and 6.971 pixels from the design, respectively; those analyzed from the samples were 1.439, 2.357, 3.198, 4.718, 5.984, and 7.093 pixels, respectively (Figure 17a). The difference between the designed and analyzed values (on average 0.564 pixels) can be attributed to reasonable fabrication tolerances. Similar statistics were also summarized for the LER on the outer contour of Arm-1 (on average 0.521 pixel (Figure 17b)), inner contour of Arm-2 (on average 0.616 pixel (Figure 18a)), and outer contour of Arm-2 (on average 0.460 pixel (Figure 18b)).

**Figure 19:**
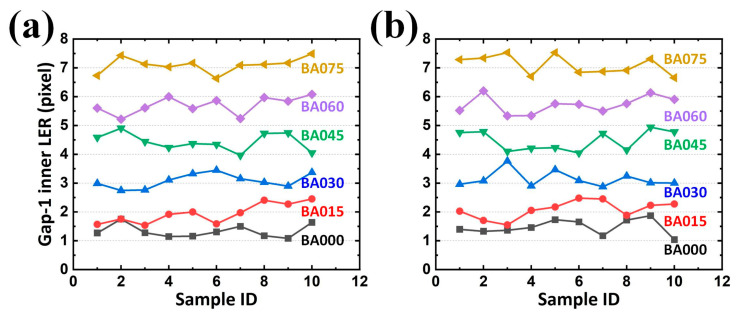
Analyzed LERs on the (**a**) inner and (**b**) outer edge of Gap-1 along various BAs in 10 PCB samples.

**Figure 20:**
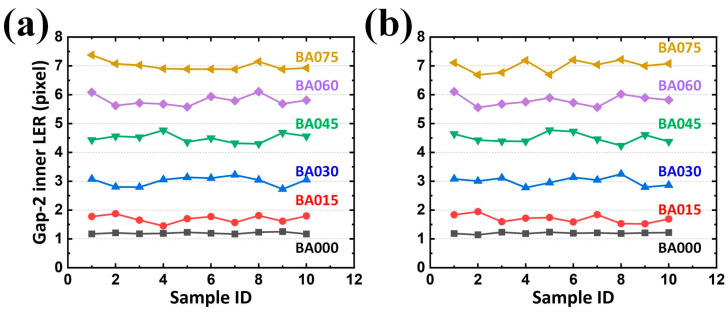
Analyzed LERs on the (**a**) inner and (**b**) outer edge of Gap-2 along various BAs in 10 PCB samples.

However, in EM applications, the responses rely on conductors (arms with metal) and insulators (gaps with dielectric) for its capacitive behavior. Two arms and their corresponding gaps were thus considered as the paired electrodes and the distance between the paired electrodes in a capacitor for a SCASA, respectively. Consequently, it is necessary to quantify the gap integrity, which is composed of two contours separately contributed by different arms. Statistically, the results revealed that an increasing average gap existed along the enlarged BA, also reflecting the expected tendency (Figure 19a,b and Figure 20a,b for LER on the inner contour of Gap-1, outer contour of Gap-1, inner contour of Gap-2, and outer contour of Gap-2, respectively). Additionally, the maximal LER in a specific BA set (e.g., 045) did not surpass the minimum LER in the adjacent BA set (e.g., 060), thereby indicating the effectiveness of the proposed procedure.

In addition to the expected differences and tendencies, the contribution of the second alignment should be further studied. When the function *Second alignment* was removed from the operation *Preprocessing*, LER analyses of the arms and gaps could still be performed. By converting the results obtained with the function *Second alignment* (Figure 17, Figure 18, Figure 19 and Figure 20) into statistical expressions and by comparing their counterparts obtained without the function *Second alignment*, the importance of the function *Second alignment* was proved.

**Figure 21:**
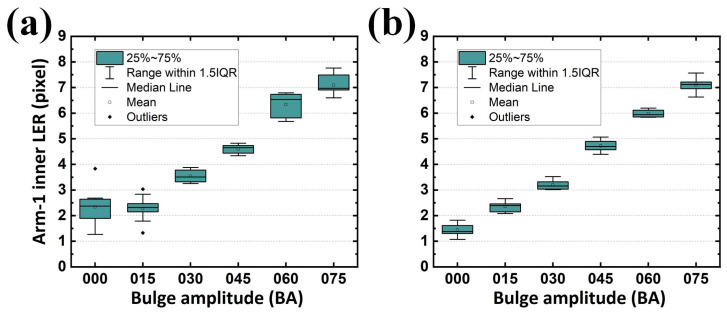
Analyzed LERs along various BAs (**a**) without and (**b**) with the proposed second alignment on the inner contour of Arm-1. IQR represents interquartile range.

**Figure 22:**
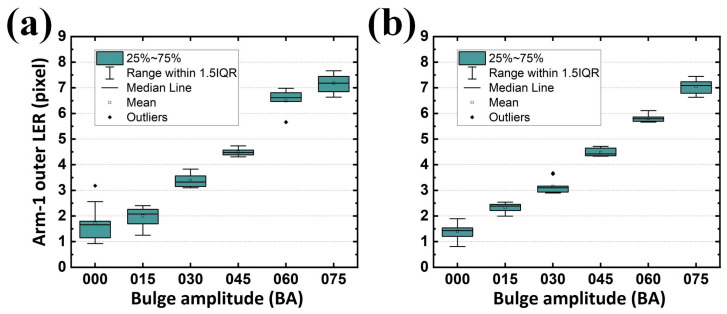
Analyzed LERs along various BAs (**a**) without and (**b**) with the proposed second alignment on the outer contour of Arm-1. IQR represents interquartile range.

**Figure 23:**
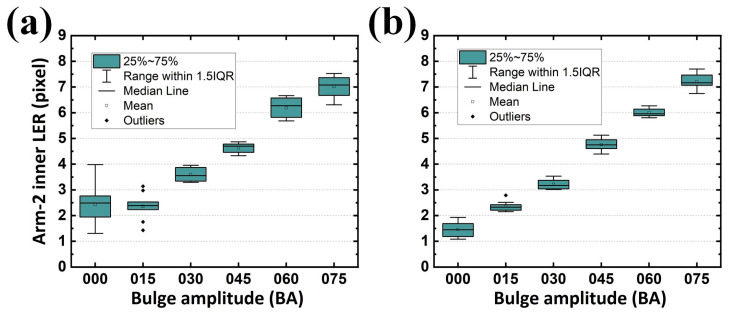
Analyzed LERs along various BAs (**a**) without and (**b**) with the proposed second alignment on the inner contour of Arm-2. IQR represents interquartile range.

**Figure 24:**
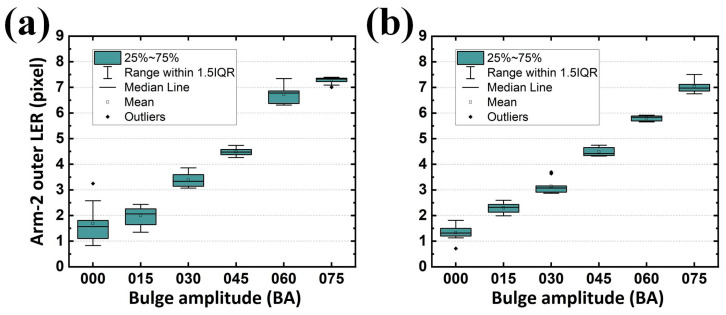
Analyzed LERs along various BAs (**a**) without and (**b**) with the proposed second alignment on the outer contour of Arm-2. IQR represents interquartile range.

When the second alignment was not performed, the LER distribution was diverse and the standard deviation was large in Arm-1 (Figure 21a). The target LER for the BA sets of 000, 015, 030, 045, 060, and 075 on the inner contour of Arm-1 were 0, 1.655, 2.894, 4.304, 5.599, and 6.971 pixels, respectively, whereas those analyzed (without the second alignment) were 2.336, 2.281, 3.539, 4.599, 6.336, and 7.093 pixels, respectively. Although the LER differences between the designed and analyzed ones (on average 0.794 pixels) may be negligible, an inappropriate LER tendency appeared in the BA sets of 000 and 015. Similarly, the statistics for the difference between the designed and analyzed (without the second alignment) LER on the outer contour of Arm-1 (on average 0.633 pixels in Figure 22a), on the inner contour of Arm-2 (on average 0.816 pixels in Figure 23a), and on the outer contour of Arm-2 (on average 0.703 pixels in Figure 24a) were summarized with an average tolerance for all four LERs of 0.737 pixels.

However, when the second alignment was conducted, the LER distribution and its standard deviation exhibited improved stability. The analyzed (with the second alignment) LERs were 1.439, 2.357, 3.198, 4.718, 5.984, and 7.093 pixels for BA sets of 000, 015, 030, 045, 060, and 075, respectively (Figure 21b). The differences between the designed and analyzed ones (on average 0.564 pixels) were improved by 29.0% compared with the results shown in Figure 21a. Similar statistics were also summarized for the difference between the designed and analyzed (with the second alignment) LER on the outer contour of Arm-1 (on average 0.521 pixels, Figure 22b, improved by 17.7%), on the inner contour of Arm-2 (on average 0.616 pixels, Figure 23b, improved by 24.5%), and on the outer contour of Arm-2 (on average 0.460 pixels, Figure 24b, improved by 34.6%). The overall average tolerance for all four LERs analyzed with the second alignment was 0.540 pixels, exhibiting a 26.7% improvement compared to its counterpart obtained without the second alignment.

Although these differences may be invisible, the standard deviation of the LER in each BA set without the second alignment was larger than that with the second alignment, implying the effectiveness of the proposed procedure. Considering that cameras in the iAOI system can be advanced for various applications, the second alignment that led to precise analysis and unambiguous judgment was crucial.

When the LERs were collected using the proposed iAOI procedure and displayed on the developed GUI, they were fed into the established AI model to predict the capacitances of the SCASAs. The results indicate that the prediction was efficient and correct, as summarized elsewhere [8], and many more features in addition to the LER may be included in the AI model in the future.

This work extended the research result from [7], which simply started with linear, symmetric, and nonlinear (but mathematically predictable) patterns. As a result, practical patterns that can be applied to applications such as antennas were developed for the first time. In addition, the designed antenna successfully imitated the inkjet printing effect of wetting. We believe that the proposed iAOI system could thus be compatible with linear, symmetric, and nonlinear, but mathematically describable patterns.

## 6. Conclusions

The spirit of this work is to provide integrated hardware and software to the operators who may not have the professional or technical background to appropriately evaluate the result. The iAOI integrates the hardware of a camera, BU, and stage, and the software of a control GUI that includes algorithms for image operation and AI-based judgment. Consequently, potential users (regardless of their background) could easily handle the production line without judgment bias. In summary, an iAOI system was built using an automatic pattern integrity analysis in this work with the following highlights.

### 6.1. An Integrated iAOI System Was Realized

In this work, a lazy learning method was applied with the help of Matlab toolbox, in which 19 models exist. We looked for a model that shows the smallest root-mean-square-error but the largest coefficient of determination as the training result. Consequently, after a thorough evaluation of all models, the Gaussian progress regression with an exponential covariance function was determined as the most suitable AI model for the iAOI system [27]. Five operations (registration, thresholding, gradient, alignment, and transformation) were thus set for this iAOI system, and several functions in each operation were prepared using Python with APIs.

### 6.2. A User-Friendly GUI Was Prepared for Easy Handling

Although some of these could be performed separately, as demonstrated in previous works, the proposed iAOI system additionally provided a user-friendly GUI, which displayed the step-by-step results of image operations. This iAOI system and procedure provides a turn-key solution to the PCB production line, where pattern variations exist regardless of its design and application.

### 6.3. Verification through a Potential PCB Production Line Was Performed

To demonstrate the effectiveness of the proposed iAOI system and procedure, proof-of-concept SCASA samples made with PCBs were introduced, and LER analyses were successfully performed regardless of geometric deformation in terms of BA. Considering the present supportiveness of our facility, it is the most balanced way to perform a proof-of-concept study. In the present case, we considered setting a reliable reference for reappearance. Nevertheless, when one of the parameters changes, it is still possible to reappear a dependable result, provided that additional studies were thoroughly carried out.

### 6.4. Additional Studies Were Conducted on the Topic of BU, Marker, and Offset

In addition, physical features of widths and gaps were quantified for the SCASA samples, and advances in sharpness enhancement, marker modification, and alignment refinement through BU improvement and diffuser optimization, triangle marker introduction, and offset elimination, respectively, were proposed and performed to further perfect the analyses.

### 6.5. Improved Accuracy and Efficiency Was Demonstrated

The results indicated that, on average, an LER accuracy improvement of 26.7% was achieved. Because the proposed iAOI system and procedure integrated the required operations into a single GUI and provided corresponding responses to the results of each operation, the analysis efficiency of complex patterns with handling simplicity substantially improved. Based on identical hardware and environmental conditions, the required operation time was suppressed from 55 min [4] to 8 min for analyzing one SCASA sample, displaying an 85% improvement in practice. The proposed iAOI system and procedure showed the capability of function extension for EM characterization and its prediction by linking the LERs to an AI model, implying its practicality in smart manufacturing.

## Data Availability

Data are contained within the article.

## Supplementary Materials

The following supporting information can be downloaded at: https://www.mdpi.com/article/10.3390/s24051619/s1, Figure S1: Draft of front-end operation structure; Figure S2: The login page; Figure S3: Work page and states panels; Figure S4: Operating flow; Figure S5: The f(t) curve when normal and (b) after derivative of Laplacian; Figure S6: Derivative value at the edge is 0 in Laplacian; Figure S7: The direction of the gradient in (a) 0°, (b) 45°, (c) 90°, and (d) 135°.

## Nomenclature

PCB	Printed circuit board	BA	Bulge amplitude
AOI	Automatic optical inspection	AI	Artificial intelligence
LWR	Line width roughness	BU	Backlight unit
LER	Line edge roughness	API	Application programming interface
EM	Electromagnetic	OM	Optical microscope
iAOI	Integrated AOI	SMD	Surface-mounted device
GUI	Graphical user interface	LED	Light-emitting diode
SCASA	Self-complimentary Archimedean spiral antenna		
