# Supplementary material for: Integrated Automatic Optical Inspection and Image Processing Procedure for Smart Sensing in Production Lines
Source: Sensors (Basel). 2024 Mar 1;24(5):1619. doi: 10.3390/s24051619 (PMC10933995; doi:10.3390/s24051619)
Supplement: Supplementary file 1 [file sensors-24-01619-s001.zip › sensors-2854595-supplementary.pdf]

Composition of the GUI (software front-end)

1. Structure

Programming language: Python (version: 3.6.8)

Package: Tkinter [SR1], os, time, threading, PIL [SR2]

Other tools: diagram.net

From the draft of GUI (Fig. S1), there are 3 main parts: login page (Fig. S2), work page (Fig. S3), and states panel (Fig. S3). After completing the password verification on the login page, it is going to jump to next frame that contains work page and states panel. Each part runs independently and has its own thread to prevent lag during working. After all the work done, it will terminate all the threads to ensure the memory is empty before next new project.

[SR1] https://docs.python.org/3/library/tkinter.html

[SR2] https://pillow.readthedocs.io/en/stable/

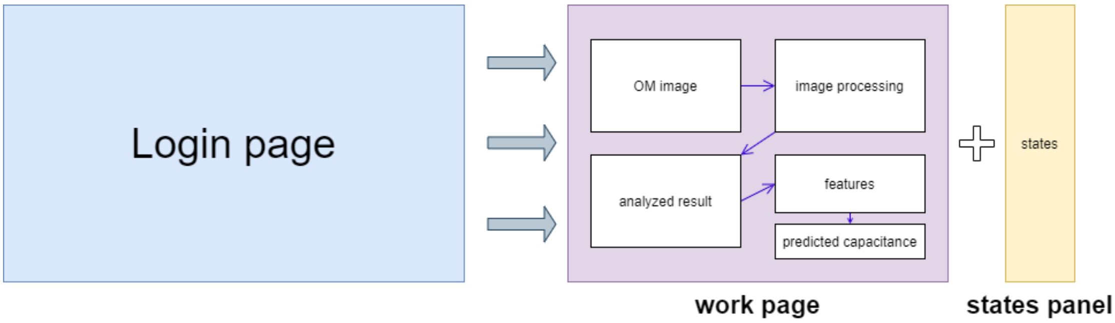

Fig. S1. Draft of front-end operation structure.

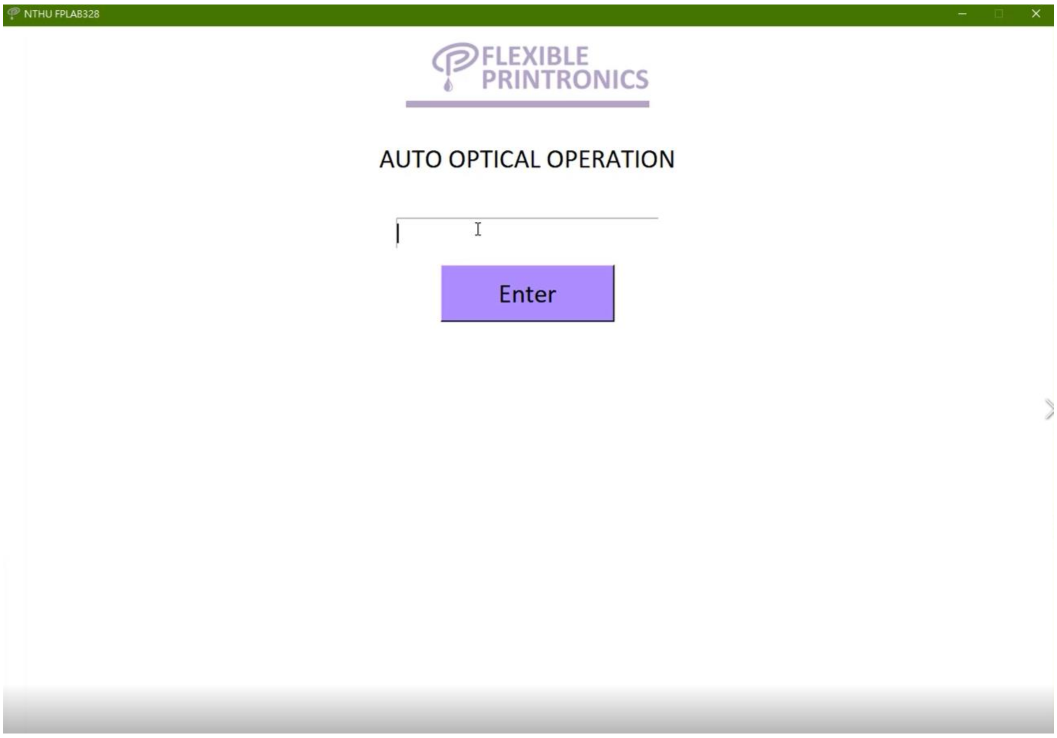

Fig. S2. The login page.

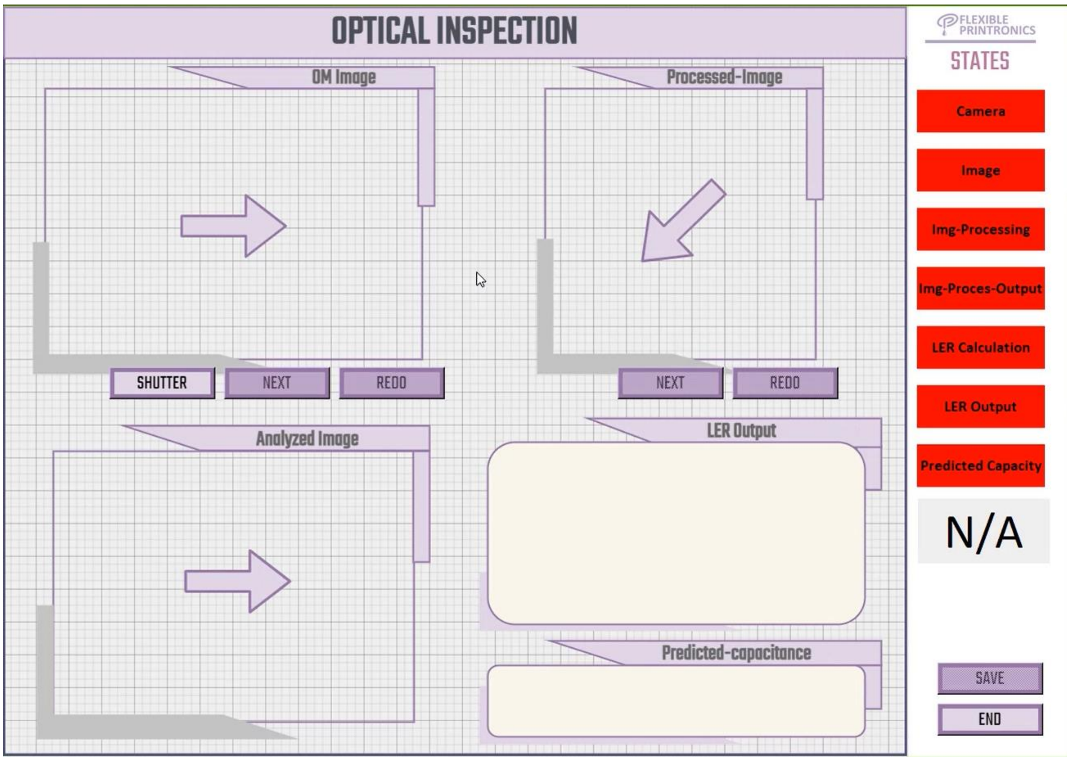

Fig. S3. Work page and states panels.

2. Logic

In the front-end operation part, when the user opens the software EXE, the login page (Fig. S2) first appeared. After verifying the password and the password was correct, the page jumped to the work frame (Fig. S3), which contained the work page and states panel. Because the two pages monitored each other during operation (Fig. S4), both pages were placed in the same frame. Moreover, because the back-end computing volume of this software was huge, it was avoided that the front-end got stuck or even crashed due to insufficient memory during the back-end computing process.

This research developed in the form of separation of the front-end and back-end. That was, the multi-threaded way gave the front-end and the back-end their respective threads. In the thread part of the front-end, the work page and states panel had their own independent threads, so that they can only monitor each other without interfering with each other. In addition, the "in progress" status and percentage progress displayed in the states panel, these two independent functions also needed to have a separate thread for control, so two more lines needed to be added to the threads of the states panel.

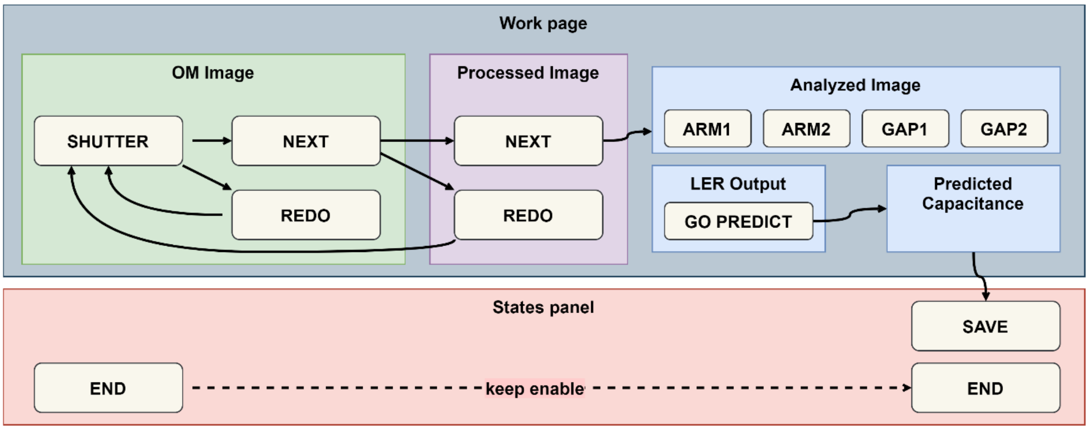

Fig. S4. Operating flow.

3. Flow

After verifying the password, press "Enter", if the password was correct, the page jumped to the work frame, in the work frame, the work page and states panel were displayed. In this step, only the SHUTTER button of the work page and the END button of the States panel were enabled.

Press SHUTTER to start the shutter of the camera, the captured image was thus displayed in the position of OM image, and the NEXT and REDO buttons of the OM image block were activated at the same time.

Press the REDO button, for removing the OM image. The SHUTTER button was reactivated, and the NEXT and REDO buttons were disabled.

Press the NEXT button, for disabling the NEXT and REDO buttons of the OM image block, and then started the Processed Image block.

After the processing was completed, the final image output will be projected to the Processed Image block, and the NEXT and REDO buttons of the block were activated.

Press the REDO button, the OM image, and Processed Image images were removed, the SHUTTER button was reactivated, and the NEXT and REDO of the OM Image and Processed Image blocks were disabled.

Press the NEXT button, disabling the NEXT and REDO buttons in the Processed Image block, starting LER calculation. The graph of contours of Arm-1, Arm-2, Gap-1, Gap-2 were displayed in the Analyzed Image block and the corresponding buttons were created. User could use the corresponding button to view each graph. In the LER Output block, the 9 feature values were displayed in Arm-1, Arm-2, and classified respectively.

Press the GO PREDICT button, starting the Predicted Capacitance block. After calculation, the capacitance values of Arm-1 and Arm-2 were displayed in the predicted Capacitance block. At the same time, the SAVE button in the States Panel was activated.

Press the SAVE button, storing the analysis data and results to the designated

folder.

Image gradient

Gradient is an operation of differentiation. Gradient can be used to find the boundary of the image [SR3]. Usually, the image is grayed out first, and the boundary has a large difference in pixel changes. For example, there must be a boundary between adjacent continuous black pixels (value defined as 0) and white pixels (value defined as 255). The greater the difference in the values of adjacent pixels, the more obvious the boundary exists. The change of adjacent and continuous pixel values can be regarded as a function, and the possible boundary formed by Gradient can be found in literature [SR4].

1. Sobel

Technically, it is a discrete difference operator used to calculate the approximate value of the gradient of the image brightness function. Using this operator at any point of the image, the operation will generate the corresponding gradient vector or its norm. Conceptually, the operator is a small integer filter that convolves the entire image in the horizontal and vertical directions, so it requires relatively few computing resources [SR5].

On the other hand, for the frequency in the image where the change is higher, the approximate value of the gradient it obtains is also relatively rough. Sobel operator is a combination of Gaussian smoothing and differential operation, thus its anti-noise ability performs well [SR6].

For example, let A represent the original image, and G_x and G_y represent the images detected by the horizontal and vertical edges respectively. The formula is defined as follows [SR7].

$$G_x = \begin{bmatrix} +1 & 0 & -1 \\ +2 & 0 & -2 \\ +1 & 0 & -1 \end{bmatrix} * A \text{ and } G_y = \begin{bmatrix} +1 & +2 & +1 \\ 0 & 0 & 0 \\ -1 & -2 & -1 \end{bmatrix} * A \quad \text{(R - 1)}$$

The approximate value of the horizontal and vertical gradient of each pixel of the image can be combined with the following formula to calculate the magnitude of the gradient.

$$G = \sqrt{{G_x}^2 + {G_y}^2} \quad \text{(R - 2)}$$

The following formula is thus used to calculate the gradient direction.

$$\theta = \arctan\left(\frac{G_x}{G_y}\right) \quad \text{(R - 3)}$$

Taking the vertical edge as an example, if the angle θ is equal to zero, it means that the

right side of the vertical edge of the image is brighter; if it is π, the left side is brighter.

2. Scharr

Scharr is the optimization version of Sobel when the kernel core 3×3. The 3×3 Scharr filter convolution kernel is defined as follows.

$$G_x = \begin{bmatrix} -3 & 0 & +3 \\ -10 & 0 & +10 \\ -3 & 0 & +3 \end{bmatrix} \quad (R-4)$$

$$G_y = \begin{bmatrix} -3 & -10 & -3 \\ 0 & 0 & 0 \\ +3 & +10 & +3 \end{bmatrix} \quad (R-5)$$

3. Laplacian

From Fig. S5, in the edge area of the image, the pixel value will jump, and the derivative of these pixels will be the extreme value at the edge position of the first derivative. This is the principle used by the Sobel operator: the extreme value is the edge. When second derivative is performed, user will find that the derivative value at the edge is 0 in Fig. S6.

The Laplace function is implemented by firstly using the Sobel operator to calculate the second-order x and y derivatives, and then get the sum, as mentioned in (R-6):

$$\text{Laplace}(f) = \frac{\partial^2 f}{\partial x^2} + \frac{\partial^2 f}{\partial y^2} \quad (3-6)$$

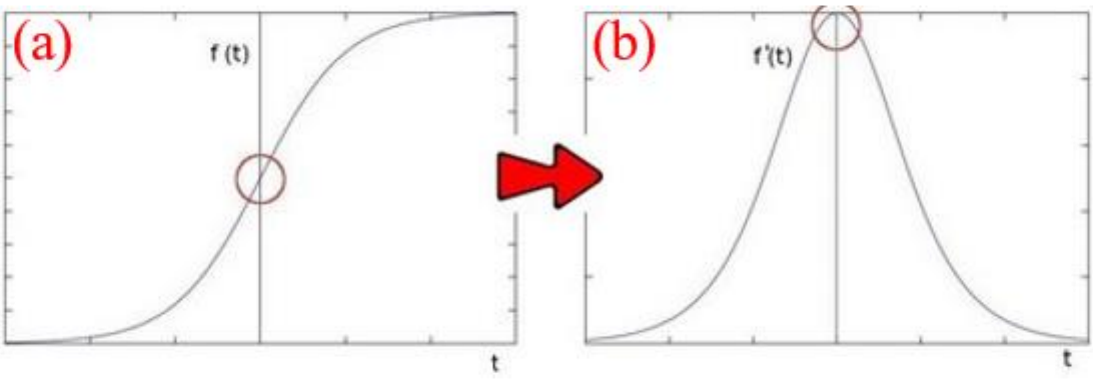

Fig. S5. The f(t) curve when normal and (b) after derivative of Laplacian [SR8].

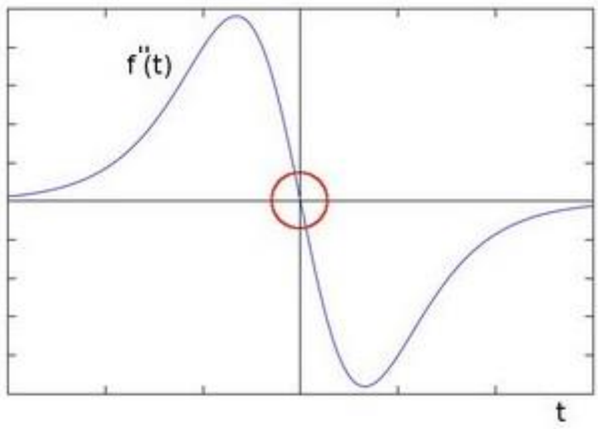

Fig. S6. Derivative value at the edge is 0 in Laplacian [SR8].

[SR3] Q. Song *et al.*, *2018 Data Compression Conference*, 2018, pp. 426-426.
[SR4] H. Wang *et al.*, *2006 International Conference on Image Processing*, 2006, pp. 2893-2896.
[SR5] J. -P. Hong *et al.*, *2020 IEEE International Conference on Consumer Electronics - Asia (ICCE-Asia)*, 2020, pp. 1-3.
[SR6] H. Zhang *et al.*, *2012 International Conference on Computer Science and Service System*, 2012, pp. 238-241.
[SR7] https://docs.opencv.org/3.4/d2/d2c/tutorial_sobel_derivatives.html
[SR8] https://docs.opencv.org/3.4/d5/db5/tutorial_laplace_operator.html

4. Canny

Canny is a composite edge detection algorithm that combines the four algorithms of Gaussian filter, gradient detection, non-maximum suppression, and boundary judgment to practice edge detection [SR9]. Its flow contains four steps.

Step1, Using Gaussian filter to filter out the noise with (R-7). The following is a Gaussian matrix with Kernel = 5 [SR10].

$$K = \frac{1}{159}\begin{bmatrix} 2 & 4 & 5 & 4 & 2 \\ 4 & 9 & 12 & 9 & 4 \\ 5 & 12 & 15 & 12 & 5 \\ 4 & 9 & 12 & 9 & 4 \\ 2 & 4 & 5 & 4 & 2 \end{bmatrix} \qquad \text{(R - 7)}$$

Step2, finding the intensity gradient of the image by Sobel.

Step3, finding non-maximum suppression. The direction of the gradient is roughly divided into four kinds, 0°, 45°, 90°, 135°. By using the maximum suppression algorithm, it is able to find the point where the gradient of the direction changes the most [SR11]. For example: it has been determined that the gradient direction is 45 °,

and the point in the matrix with the highest intensity in the direction of 45° is gradually searched for, and the remaining points in the direction of 45° are all returned to 0 [SR12].

Step4, connecting weak edge. Setting the high and low boundaries and following this rule to find the edge.

From Fig. S7, There are three different situations. (a) Above the high boundary, it must be the edge; (b) below the low line, it must not be the edge; and (c) between the high boundary and the low boundary, this point is also regarded as an edge if there are two nearby points higher than the high boundary [SR8].

Additionally, Canny shows the following advantages.

A. Low error rate. Most of the detected bright spots are edges.
B. Accurate positioning. The marked edge is close to the actual edge.
C. High resolution. Fine lines on the edges.

About the function of canny, there are only 3 parameters, which are apertureSize, threshold1 and threshold2, can be adjusted. The options of apertureSize are 1, 3, 5, 7. The range of threshold1 and threshold2 are both from 0 to 255. As a result, there are (4×255×255) = 260,100 combinations. Finally, users can find out that the combination of apertureSize = 7, threshold 1 = 90, threshold 2 = 255 performs the best result, thus deciding to use this combination in the image processing [SR13].

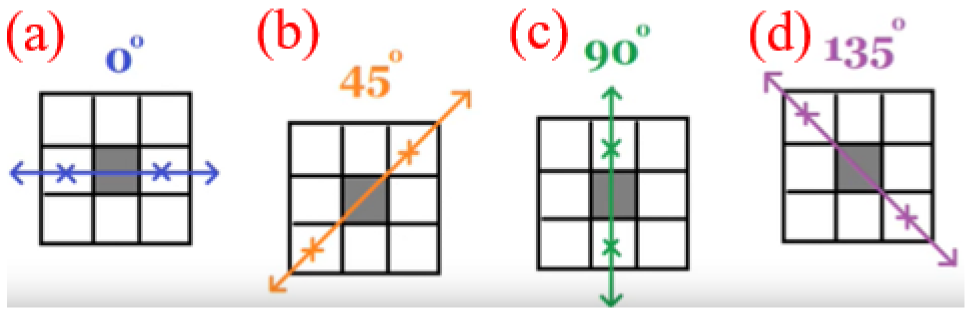

Fig. S7. The direction of the gradient in (a) 0°, (b) 45°, (c) 90°, and (d) 135° [SR10].

[SR9] J. G. Thanikkal *et al.*, *2018 7th International Conference on Reliability, Infocom Technologies and Optimization (Trends and Future Directions) (ICRITO)*, 2018, pp. 1-5.
[SR10] https://docs.opencv.org/3.4/da/d22/tutorial_py_canny.html
[SR11] R. Dhar *et al.*, *2014 International Conference on Green Computing Communication and Electrical Engineering (ICGCCEE)*, 2014, pp. 1-6.
[SR12] A. Bera, *2011 3rd International Conference on Electronics Computer Technology*, 2011, pp. 164-167.
[SR13] T. Batard *et al.*, *2009 16th IEEE International Conference on Image Processing (ICIP)*, 2009, pp. 461-464.

Supplementary file (supporting information)
